# Examining the Effect of Polypharmacy on Quality of Life and Frailty in Older Adults from the Perspective of Community-Based Rehabilitation

**DOI:** 10.3390/healthcare13131531

**Published:** 2025-06-27

**Authors:** Mustafa Cemali, Aynurhayat Kanlıca, Sıla Yılmaz, İlayda Yılmaz, Özgün Elmas, Aynur Ayşe Karaduman

**Affiliations:** 1 Faculty of Health Sciences, Department of Occupational Therapy, Trakya University, 22030 Edirne, Turkey; 2 Faculty of Health Sciences, Department of Occupational Therapy, Lokman Hekim University, 06530 Ankara, Turkey; kanlicaaynul@gmail.com (A.K.); silaa.yilmmaz@gmail.com (S.Y.); ilayda00245@gmail.com (İ.Y.); ayse.karaduman@lokmanhekim.edu.tr (A.A.K.); 3 Faculty of Health Sciences, Department of Physiotherapy and Rehabilitation, Siirt University, 56100 Siirt, Turkey; ozgunelmas@hotmail.com

**Keywords:** polypharmacy, older adult, quality of life, frailty, community-based rehabilitation

## Abstract

**Objective:** Although the negative effects of polypharmacy on older adults are well-documented, studies exploring its relationship with frailty and quality of life within the framework of community-based rehabilitation (CBR) remain scarce. In this context, the aim of this study was to compare frailty and quality of life levels between older adults with and without polypharmacy and to examine the relationship between these parameters from a CBR perspective. The ultimate purpose of this study was to determine the usefulness of CBR. **Method**: A total of 120 community-dwelling older adults (60 with polypharmacy, 60 without polypharmacy), aged 65–75 years (mean age = 68.18 ± 3.50), were included in a community-based assessment carried out under the coordination of Lokman Hekim University in Ankara, Turkey. The use of five to nine medications was taken as a reference for those with polypharmacy, and the use of less than two medications was taken as a reference for those without polypharmacy. The quality of life of the older adults in the study was assessed with the Nottingham Health Profile (NHP), and frailty was assessed with the Edmonton Frailty Scale (EFS). In line with CBR principles, the findings were interpreted with a focus on promoting community-wide strategies to support older adults. **Results:** The study found a statistically significant difference in NHP and EFS results between older adults with and without polypharmacy (*p* < 0.05). In addition, a statistically significant relationship was found between NHP and all subdomains of NHP and EFS (*p* < 0.05). **Conclusion:** Older adults with polypharmacy had higher levels of frailty and lower quality of life, and an increase in frailty was significantly associated with a decrease in quality of life in both groups. These findings highlight the importance of community-level preventive interventions to support healthy aging. Within the framework of CBR, strategies such as creating accessible physical activity areas at the neighborhood level; organizing informative seminars on frailty, quality of life, medication use and health literacy in collaboration with volunteer health professionals and local authorities; and creating volunteer support networks to increase social interaction can contribute to the control of these symptoms in older adults.

## 1. Introduction

Polypharmacy is defined as the concurrent use of multiple medications and is generally associated in the literature with the use of five or more drugs [1]. In some sources, this condition is classified in more detail: the use of 5–9 drugs is defined as polypharmacy, and the use of 10 or more drugs is referred to as hyperpolypharmacy [2,3]. Polypharmacy is particularly common in older adults, and its prevalence in this group is reported to range from 23% to 39% [4]. As one of the geriatric syndromes, polypharmacy negatively affects quality of life and functionality; it increases morbidity and mortality rates and can lead to multiple system disorders [5,6].

The concept of quality of life is defined as meeting all the needs of the individual; being satisfied with life; being in a good cognitive, emotional and physical state; and being able to maintain relations with the environment [7]. In various studies examining the relationship between polypharmacy and quality of life, it has been reported that the symptomatic effects of polypharmacy negatively impact both the physical health-related quality of life and the psychosocial well-being of older adults [8,9].

Polypharmacy is considered an important factor that increases the risk of frailty in older adults. The concurrent use of multiple medications can cause problems such as falls, loss of appetite, fatigue and cognitive effects, thereby reducing the individual’s overall resilience [10]. Studies have shown that this condition makes older individuals more vulnerable to stressors and may play a role in increasing frailty levels [11,12].

The older adult population is increasing worldwide, and therefore understanding the health problems associated with old age, reducing their impact and taking preventive measures before they become ill is of paramount importance for the sustainability of the healthcare system [13]. In this context, the World Health Organization’s 2021–2030 “Decade of Healthy Ageing” strategic action plan aims to protect the health of older individuals and support their social participation [14]. Parameters such as balanced nutrition, physical activity and social participation support healthy aging [15]. These areas can be supported by person-centered intervention approaches based on the needs and wishes of the person, but this may be insufficient to take into account environmental factors in sustaining health and ensuring the participation of older adults in activities [16]. Community-based rehabilitation (CBR) approaches that create opportunities for individuals in the community and identify central and local governments, health professionals, associations, non-governmental organizations, foundations and every component of society as stakeholders help individuals to participate in activities in all areas of life [17]. In this respect, the medical model, which was initially preferred in approaches to disease, has started to be supported by person-centered and CBR approaches [18].

A review of the literature shows that studies conducted within the scope of CBR in older adults include multidimensional interventions such as supporting physical activity, increasing social participation, providing home care and counseling services, and strengthening interaction with the community. These practices have been reported to contribute to the maintenance of functional independence, reduction of social isolation and support of psychological well-being in older individuals [19,20,21]. However, studies that simultaneously examine the relationship between polypharmacy and frailty in older adults are limited [22,23]. Especially in older adults with and without polypharmacy, studies that evaluate these two parameters together are lacking, and current research generally handles these variables independently. Moreover, no original study in the literature has been found that comprehensively examines this relationship within the framework of the CBR approach. Based on this, the aim of our study was to compare quality of life and frailty in older adults with and without polypharmacy and to examine the relationship between these parameters from the perspective of CBR. The ultimate purpose of this study was to determine the usefulness of CBR.

## 2. Material and Methods

### 2.1. Study Design

This cross-sectional observational study was conducted between December 2024 and March 2025 within the Department of Occupational Therapy at Lokman Hekim University, located in Ankara, Türkiye. The study was carried out with the approval of the Lokman Hekim University Scientific Research Ethics Committee (Ethical Approval Number: 2024032, dated 29 November 2024).

Older adults aged between 65 and 75 years, living independently in the community, were included in the study and categorized into two groups based on their polypharmacy status. Participants with and without polypharmacy were recruited using the snowball sampling method. This non-probability sampling technique relies on existing participants to refer new participants from their social networks and enabled the identification of eligible individuals through community-based resources such as local senior centers, neighborhood solidarity groups and social activity clubs [24]. Due to its dependence on social networks, the snowball sampling method carries a risk of selection bias. To mitigate this potential limitation, multiple independent starting points were used, and participants were recruited from diverse neighborhoods and social environments to enhance sample representativeness [25].

Furthermore, the findings of this study were planned to be interpreted within the framework of CBR. This approach aims to identify the health-related needs of older adults and to develop protective and empowering strategies at the community level to support quality of life and reduce frailty [19,20,21].

Written informed consent was obtained from all individuals who agreed to participate in the study. The research was conducted in accordance with the principles of the Declaration of Helsinki, and the study report was prepared following the Strengthening the Reporting of Observational Studies in Epidemiology (STROBE) guidelines [26].

### 2.2. Participants

Polypharmacy is defined as the use of more than one medication (number of medications ≥2) concurrently; however, the literature provides various threshold ranges to define this concept more precisely [27]. According to widely accepted classifications, the use of five to nine medications (5 ≤ number of medications ≤ 9) is considered polypharmacy, whereas the use of ten or more medications (number of medications ≥ 10) is referred to as hyperpolypharmacy [2,3]. This classification is supported by evidence indicating that an increasing number of medications is associated with potential adverse effects, drug–drug interactions and negative health outcomes such as reduced functional capacity [28]. In the present study, based on these commonly cited thresholds, individuals taking zero or one medication (number of medications ≤ 1) were classified as older adults without polypharmacy, while those taking five to nine medications (5 ≤ number of medications ≤ 9) were classified as older adults with polypharmacy.

The inclusion criteria for older adults with polypharmacy were as follows: being between 65 and 75 years of age, taking five to nine medications (5 ≤ number of medications ≤ 9) [2], being able to walk independently, and scoring 24 or above on the Mini Mental State Examination (MMSE) [29]. For older adults without polypharmacy, the inclusion criteria were as follows: age between 65 and 75 years, taking zero or one medication (n ≤ 1) [27], independent ambulation and an MMSE score of ≥24 [29]. The exclusion criteria for all participants were as follows: having an uncontrolled chronic disease other than polypharmacy that is uncontrolled and affects functionality; having neurologic, rheumatologic, oncologic, serious cardiovascular or respiratory diseases, or a history of active cancer; having received chemotherapy; being in the recovery phase of an acute disease; and having a history of surgery in the last 6 months.

The research sample consisted of older adults with and without polypharmacy who volunteered to participate in the study. The sample size of the planned research was calculated by a priori-type power analysis by selecting the statistical test of comparing the means of two independent groups (2 groups), utilizing the one-tail test parameter from the *t*-test family using the G power (3.1.9.6 version) program. In the analysis based on the Nottingham Health Profile, assuming a medium effect size (effect size = 0.5), a 95% confidence interval, and 80% statistical power, the minimum required sample size was calculated as 110 participants (55 older adults with polypharmacy, 55 older adults without polypharmacy). In the study, 126 older adults (64 older adults with polypharmacy, 62 older adults without polypharmacy) were evaluated, but 6 people (4 older adults with polypharmacy, 2 older adults without polypharmacy) were excluded because they could not complete the evaluations, and the study was completed with 120 older adults (60 older adults with polypharmacy, 60 older adults without polypharmacy).

### 2.3. Data Collection Tools

A sociodemographic form was used to collect information on age (years), gender, height (centimeters), body weight (kilograms), disease status and number of medications used in both groups of older adults participating in the study. The height and weight measurements of the older adults were directly taken by the researchers in accordance with standard procedures. Age and gender information was recorded based on official identification documents, while disease status and the number of medications used were documented using hospital reports and current prescriptions provided by the participants. The participants’ quality of life was then assessed using the Nottingham Health Profile, and frailty status was assessed using the Edmonton Frailty Scale. The assessment results for both groups were compared, and the relationship between these results was analyzed. The assessments were performed by physiotherapists and occupational therapists and took an average of 25 min.

#### 2.3.1. Nottingham Health Profile (NHP)

The Nottingham Health Profile (NHP) used to assess quality of life was developed by Hunt et al. [30]. Assessment of the Turkish reliability and validity of the NHP was conducted by Küçükdeveli et al. (reliability coefficient = 0.88, internal consistency coefficient = 0.87) [31]. The NHP consists of 38 questions and six subsections: energy (3 items), pain (8 items), emotional reactions (9 items), sleep (5 items), social isolation (5 items) and physical activity (8 items). Each subsection ranges from 0 to 100 points, and the total score ranges from 0 to 600. Higher scores indicate more limitations on quality of life [31].

#### 2.3.2. Edmonton Frailty Scale (EFS)

The EFS was developed by Rolfson et al. in 2006 to assess frailty in older adults [32]. The scale comprises 11 items based on the Likert scale, which evaluate various factors in older adults, including drug use, cognitive status, social support, general health status, functional independence, mood, continence, functional performance and nutrition. The total score varies between 0 and 17 points, and a higher score indicates increased frailty. The validity and reliability of the scale was determined by Aygör et al., and the Cronbach’s alpha coefficient was found to be 0.75 [33].

### 2.4. Data Analysis

The SPSS Statistics IBM Version 26.0 program was used for statistical analyses of the data. The conformity of the acquired data to normal distributions was analyzed by Skewness, Kurtosis Histograms and the Kolmogorov–Smirnov Test. As a result of the analysis, it was determined that categorical variables were not normally distributed and that numerical variables were normally distributed. The chi-square test was used in the comparison of categorical variables, and the data were presented as numbers (n) and percentages (%). The Independent-Samples *t*-Test was used for the comparison of numerical variables, and the data were presented as means ± standard deviations (means ± SDs). In addition, Cohen’s d was calculated to determine the effect size of group differences based on the Independent-Samples *t*-Test results. Effect size benchmarks were determined as <0.30, 0.30–0.80 and >0.80 and considered small, moderate and strong, respectively [34]. The relationship between the evaluations was analyzed by the Pearson correlation test. Statistical significance was accepted as *p* < 0.05.

## 3. Results

Information about the participants’ gender, diseases, age, height, weight and number of medications used is given in Table 1. A total of 120 older adults aged 65–75 years (60 older adults with polypharmacy and 60 older adults without polypharmacy) with a mean age of 68.18 ± 3.50 years participated in the study. There was no statistically significant difference between the groups in terms of gender, age, height or weight (*p* > 0.05). There was a statistically significant difference between the groups with polypharmacy (number of drugs between five and nine) and without polypharmacy (number of drugs between zero and one) in terms of the number of drugs used (*p* < 0.001).

Statistically significant differences were found between the groups for NHP, NHP subdomain and EFS results (*p* < 0.001). It was found that older adults with polypharmacy had worse general quality of life than older adults without polypharmacy and scored worse in all sub-fields of quality of life. It was found that older adults with polypharmacy had a higher level of frailty than older adults without polypharmacy (Table 2).

The results for the relationship between frailty and NHP and NHP subdomains in older adults are given in Table 3. A statistically significant relationship was found between frailty and NHP and NHP subdomains in both groups (*p* < 0.05). It was observed that frailty increased with worsening pain levels, physical activity levels, sleep status, social isolation and emotional reactions, which are sub-fields of quality of life and quality of life in older adults with and without polypharmacy (Table 3).

## 4. Discussion

According to the results of the study, older adults with polypharmacy were found to experience more problems compared to those without polypharmacy in terms of frailty, overall quality of life and specific subcomponents of quality of life, such as pain, physical activity, energy, sleep, social isolation and emotional responses. Furthermore, a deterioration in overall quality of life and its subdomains was found to be associated with increased frailty.

Polypharmacy disrupts the physiological functioning of the body due to the interactions among the active ingredients of medications [35]. This physiological disruption can lead to impairments in several functional domains, including cognitive, physical, emotional and mental health domains [5]. Liu et al. reported that deficits in these domains, which are often associated with polypharmacy in the literature, contribute to increased frailty in older adults [22]. Similarly, Fernandez et al. demonstrated that polypharmacy may lead to musculoskeletal problems in older individuals, such as pain, loss of balance and muscle weakness [36]. Another study found that multiple medication use has negative effects on the psychosocial status and sleep quality of older adults [37]. In our study, older adults with polypharmacy exhibited higher levels of frailty, lower quality of life and worse outcomes in subdomains of quality of life, such as pain, physical activity, energy, sleep, social isolation and emotional responses. Based on these findings, it can be inferred that polypharmacy adversely affects both frailty and quality of life by impairing physiological system function in older adults [38]. The overlap between the domains negatively affected by polypharmacy and those that determine frailty and quality of life may explain the more pronounced deterioration observed in these areas among older individuals [9]. However, it should be noted that participants in this study had varying chronic diseases, which may also contribute to changes in quality of life and frailty. Therefore, future research should investigate the effects of polypharmacy in specific patient groups in more detail. In addition, the literature emphasizes the growing importance of CBR approaches, which are rooted in preventive strategies, alongside individual-centered medical management. CBR has shown promising results in managing disease-related impacts across different patient populations [20,39]. In line with the CBR approach, community-level awareness-raising and educational interventions are essential for the prevention and management of polypharmacy-related problems. In particular, health literacy seminars should be organized in community health centers, family health units and public education institutions. These seminars should target older adults, caregivers and family members, and address key topics such as medication use, drug interactions and the risks associated with polypharmacy [40]. The seminars should explicitly highlight the impact of polypharmacy on quality of life and frailty, and provide information on rational drug use, medication monitoring and the prevention of drug–nutrient interactions. These strategies may be effective in mitigating the negative effects of polypharmacy on quality of life and frailty [41]. In addition, strategies to prevent polypharmacy should be initiated at an early age. Health literacy programs for young adults should focus on increasing awareness regarding the inappropriate use of over-the-counter medications, dietary supplements and substances with addictive potential, as well as their long-term adverse effects. Educational initiatives implemented through universities and youth centers may help reduce the future risk of polypharmacy and contribute to the maintenance of quality of life and functional capacity [42].

Due to aging, the functioning of body systems such as the musculoskeletal, cardiovascular, cardiopulmonary, endocrine and nervous systems may deteriorate [43]. The literature indicates that as individuals age, both the number of chronic conditions and the use of medications tend to increase. Consequently, polypharmacy may lead to disruptions in bodily structures and systems, resulting in impairments in cognitive, behavioral, psychosocial, sensory and motor functions among older adults [5]. Lima et al. reported that increased cognitive, mental health and behavioral problems associated with polypharmacy reduce the quality of life in older individuals [44]. Similarly, Herr et al. identified polypharmacy-related musculoskeletal issues as a significant risk factor for frailty [45]. Another study indicated that older adults with impairments in motor skills, balance and mobility may be at risk for developing geriatric syndromes such as sarcopenia [46]. Cemali et al. demonstrated that age-related slowing of neural conduction impairs the functioning of sensory systems, contributing to problems in vision, hearing, touch and balance [47]. The literature shows that these physiological and functional changes caused by aging and polypharmacy are significant determinants negatively affecting frailty and quality of life [22]. In this study, older adults with and without polypharmacy were compared, and it was found that decreases in quality of life were accompanied by increases in frailty levels. The findings suggest that the relationship between quality of life and frailty is not only based on shared determinants but also reflects a dynamic interaction between the two. Therefore, multidisciplinary and holistic intervention strategies are essential for improving both indicators.

These findings suggest that polypharmacy, age-related systemic changes and geriatric syndromes may create a reinforcing cycle. Therefore, in addition to clinical monitoring, there is a clear need for protective and community-level strategies. In this context, the CBR approach stands out as an effective and multidimensional framework that supports the physical, social and emotional well-being of older adults, contributing both to the enhancement of quality of life and the reduction of frailty risk. The CBR model can be implemented not only through individual-level interventions but also by promoting community-based awareness activities and accessible opportunities in areas directly affecting older adults, such as physical activity levels, nutritional quality and social interaction [19]. Collaboration between local and national governments to develop walking paths, indoor/outdoor sports areas and parks near residential areas may encourage regular physical activity habits [17]. In healthy aging centers, structured group exercise sessions, including balance and coordination training (e.g., core stabilization, calisthenics and breathing exercises), should be offered under professional supervision. In addition, education programs delivered by health professionals should cover topics such as disease management, medication use, physical activity, pain control, joint and energy conservation techniques, mood regulation and psychosocial support [48]. Nutrition education for older adults should address weekly menu planning, portion control and adequate protein intake. To support sleep quality, sleep hygiene guidance should be provided, including recommendations such as avoiding heavy evening meals and limiting caffeine intake, along with behavioral strategies (e.g., ending physical activity in the early evening, reducing screen exposure and establishing regular sleep hours) [49]. To ensure the effectiveness of these practices, regular informational meetings should be organized for older adults, caregivers and family members through collaboration among health professionals, the Ministry of Health and local governments [50]. Furthermore, multipurpose healthy living centers should be established where older adults can participate in leisure activities such as cinema, theater, choir and crafts, and where community seminars can also be conducted. Programs promoting intergenerational interaction should be included in these centers to strengthen older adults’ social ties and increase community participation [51]. Information on these topics should be delivered to older adults via brochures, posters and visual and written media, as well as through community leaders and local authorities. Public service announcements and educational campaigns should be used to increase societal awareness. Through these strategies, quality of life can be improved, frailty risk can be reduced and the adverse effects of chronic diseases can be minimized, thereby supporting healthy and high-quality aging processes [52,53]. Looking ahead, health literacy programs developed in line with the CBR approach for younger individuals are also of great importance. These programs should focus on topics such as regular physical activity, balanced nutrition, sleep hygiene, rational drug use and awareness of addictive substances. Instilling these healthy lifestyle habits from an early age may have a protective effect by reducing future risks of polypharmacy, improving quality of life and preserving functional capacity [53].

This study has several limitations. Most older adults in the polypharmacy group had multiple chronic diseases, which may weaken the ability to attribute the observed associations between frailty and quality of life solely to polypharmacy. Additionally, the participants were selected from a single geographical region, which limits the generalizability of the findings to different cultural, socioeconomic and healthcare contexts. On the other hand, studies conducted in diverse regions could offer not only broader generalizability but also valuable insights for CBR practices. Regional variables such as physical environmental conditions, access to social services and the structure of the healthcare system are crucial determinants of the design and effectiveness of CBR interventions. Therefore, future research should include multicenter and longitudinal studies conducted in different geographical and cultural settings and focused on specific disease groups. These studies would contribute to the development of flexible CBR models that are tailored to local needs and consider regional resources.

## 5. Conclusions

According to the results of the study, it was found that older adults with polypharmacy had more problems in terms of frailty, quality of life and subcomponents of quality of life such as pain, physical activity, energy, sleep, social isolation and emotional reactions compared to older adults without polypharmacy. In addition, it was determined that frailty increased with the worsening of quality of life and subdomains of quality of life such as pain, physical activity, energy, sleep, social isolation and emotional reactions. It is anticipated that the CBR method may be beneficial in keeping polypharmacy, quality of life and frailty problems under control in older adults within the scope of healthy aging. In order to understand this effect, CBR interventions should be planned with randomized controlled trials in this population and patient group.

## Figures and Tables

**Table 1:** Identifying information about participants.

	Older Adults with Polypharmacy	Older Adults Without Polypharmacy	*p*
	n = 60	n = 60	
	n (%)	n (%)	
**Gender**			
Female	28 (46.6%)	31 (51.6%)	0.584 ^a^
Male	32 (53.4%)	29 (48.4%)	
**Disease**			
Type 2 diabetes mellitus	26 (43.3%)	15 (25%)	*p* = 0.34 ^a^
Hypertension	22 (36.7%)	12 (20%)	*p* = 0.43 ^a^
Heart diseases	20 (33.3%)	9 (15%)	*p* = 0.19 ^a^
Orthopedic diseases	17 (28.3%)	8 (13.3%)	*p* = 0.043 ^a^
Other disease	10 (16.7%)	3 (5%)	*p* = 0.040 ^a^
	**Mean ± SD**	**Mean ± SD**	***p***
**Age (years)**	69.13 ± 3.68	68.50 ± 3.3	0.324 ^b^
**Weight (kg)**	77.78 ± 12.15	74.3 ± 9.94	0.082 ^b^
**Height (cm)**	162.25 ± 8.7	164.8 ± 7.99	0.097 ^b^
**Number of drugs used**	6.06 ± 1.28	0.81 ± 0.39	*p* < 0.001 ^b^

n: number of people, %: percentage. ^a^ Chi-square test; ^b^ Independent-Samples *t*-Test, *p* < 0.05.

**Table 2:** Comparison of NHP, NHP subdomain and EFS results between groups.

	Older Adults with Polypharmacy	Older Adults Without Polypharmacy		
	n = 60	n = 60		
	Mean ± SD (Score)	Mean ± SD (Score)	*p*	Effect Size (Cohen’s d)
**NHP total**	332.31 ± 120.89	189.59 ± 90.46	*p* < 0.001	1.33
Pain	57.91 ± 24.02	31.04 ± 18.33	*p* < 0.001	1.25
Physical activity	52.33 ± 25.53	33.33 ± 19.07	*p* < 0.001	0.84
Energy	58.88 ± 33.88	33.89 ± 28.45	*p* < 0.001	0.79
Sleep	52.33 ± 25.53	36 ± 20.43	*p* < 0.001	0.70
Social isolation	53.66 ± 26.16	27 ± 21.09	*p* < 0.001	1.12
Emotional reactions	53.88 ± 25.2	28.33 ± 17.88	*p* < 0.001	1.16
**EFS**	12.06 ± 2.48	8.43 ± 4.09	*p* < 0.001	1.07

NHP: Nottingham Health Profile; EFS: Edmonton Frailty Scale; n: number of people, SD: standard deviation; Independent-Samples *t*-Test, *p* < 0.05.

**Table 3:** Association between EFS and NHP total scores and subdomains for each group.

	EFS			
	Older Adults with Polypharmacy		Older Adults Without Polypharmacy	
	n = 60		n = 60	
	*p*	r	*p*	r
**NHP total**	<0.001	0.521	<0.001	0.512
Pain	<0.001	0.595	0.001	0.425
Physical activity	<0.001	0.443	0.023	0.293
Energy	0.002	0.397	0.002	0.391
Sleep	0.019	0.302	0.001	0.418
Social isolation	0.011	0.325	0.046	0.259
Emotional reactions	<0.001	0.453	<0.001	0.439

NHP: Nottingham Health Profile; EFS: Edmonton Frailty Scale; n: number of people, r: correlation coefficient; Pearson correlation test, *p* < 0.05.

## Data Availability

The data presented in this study are available on request from the corresponding author due to privacy or ethical restrictions.

## Abbreviations

cm	Centimeter
EFS	Edmonton Frailty Scale
kg	Kilogram
NHP	Nottingham Health Profile
n	Number of people
%	Percentage
WHO	World Health Organization
SD	Standard deviation
STROBE	Strengthening the Reporting of Observational Studies in Epidemiology
